# The Use of Artificial Neural Network for Prediction of Dissolution Kinetics

**DOI:** 10.1155/2014/194874

**Published:** 2014-06-16

**Authors:** H. Elçiçek, E. Akdoğan, S. Karagöz

**Affiliations:** ^1^Department of Naval Architect and Marine Engineering, Faculty of Naval Architecture & Maritime, Yildiz Technical University, 34383 Istanbul, Turkey; ^2^Department of Mechatronics Engineering, Faculty of Mechanical Engineering, Yildiz Technical University, 34383 Istanbul, Turkey; ^3^Department of Chemical Engineering, Faculty of Engineering, Texas A&M University, College Station, TX 77843-3122, USA

## Abstract

Colemanite is a preferred boron mineral in industry, such as boric acid production, fabrication of heat resistant glass, and cleaning agents. Dissolution of the mineral is one of the most important processes for these industries. In this study, dissolution of colemanite was examined in water saturated with carbon dioxide solutions. Also, prediction of dissolution rate was determined using artificial neural networks (ANNs) which are based on the multilayered perceptron. Reaction temperature, total pressure, stirring speed, solid/liquid ratio, particle size, and reaction time were selected as input parameters to predict the dissolution rate. Experimental dataset was used to train multilayer perceptron (MLP) networks to allow for prediction of dissolution kinetics. Developing ANNs has provided highly accurate predictions in comparison with an obtained mathematical model used through regression method. We conclude that ANNs may be a preferred alternative approach instead of conventional statistical methods for prediction of boron minerals.

## 1. Introduction

Natural resources play an immense role in creating the country's wealth and spurring economic growth, and they have a significant impact on the human-social development of countries [1, 2]. In addition, they play an effective role in the developed countries' technologies and level of prosperity, provide employment, satisfy energy needs and services, encourage subindustry and manufacturing, give regional development prominence, and provide foreign exchange [3]. Turkey has very diverse natural mineral deposits because of the loam and strategic position of its geographical features. Except for the petroleum and coal, there are 53 exploitable minerals and metals and 4,500 mineral deposits in Turkey [4]. Among these minerals, undoubtedly boron is the most important mineral in terms of mineral reserve and production capacity in Turkey, which has approximately 72% of the world's boron reserves and by the end of 2007 has risen to the first place in the boron market [5].

Boron is one of the most important elements in the world due to its strategic and industrial value [6]. It is frequently used in nuclear engineering, high-quality steels, production of heat resistant polymers, cosmetic, leather, ceramics, rubber, paint, textile, agricultural catalysts, and in other industries [7, 8]. It is never found free in nature but invariably occurs as the B_2_O_3_, in combination with the oxides of other elements, forming borates of greater or lesser complexity [9]. It is known that there are more than 230 boron types in the world. Among these minerals are commercially important ones: tincal (Na_2_B_4_O_7_ *·*10H_2_O), colemanite (Ca_2_B_6_O_11_ *·*5H_2_O), and ulexite (NaCaB_5_O_9_ *·*8H_2_O) [10, 11].

Boron mineral products, which contain impurities, are comprised mostly of clays and other constituent minerals that tend to be associated with clays [12]. Dissolution processes are applied to overcome these negative effects or to obtain product in industry.

Dissolution of the mineral is related to typical industrial processes, such as those in hydrometallurgy, medicine, oceanography, crystallography, ceramics, and desalination. On the other hand, it is also used for biological and environmental precipitation processes [13].

Colemanite, which is one of the most important boron minerals, is currently used on a large industrial scale. The upswing in the demands for minerals will continue in the years ahead as they are directly linked to rapid development of the technology. The increasing demand and new industrial use of boron compounds have increased their importance, and these compounds have been used as raw material in various areas of industry. There are numerous studies in the literature about dissolution of boron in order to meet the industry demands. Some of these studies are shown in Table 1. Statistical and numerical methods were usually used to determine dissolution kinetics in these studies.

Some obtained mathematical models are given as follows: Çopur et al. studied dissolution kinetics of colemanite in water saturated with carbon dioxide solutions. They have obtained a mathematical equation as follows by using statistical method [14]:

(1) x=1−exp⁡(−6890×P0.18×KS−1.31      × TB−1.04exp⁡⁡(−2764T)×z0.47),

where *P*, *KS*, *TB*, *T*, and *z* indicate total pressure, solid/liquid ratio, particle size, temperature, and reaction time, respectively. Guliyev et al. studied colemanite in potassium hydrogen sulphate solutions. They used heterogeneous reaction models to determine the correlation between the dissolution rate and the parameters. Model equation was determined using both numerical and analytical methods as below [8]:

(2) 1−3(1−x)2/3+2(1−x)  =10.41×C1.01×W1.55×D−1.43   ×(SL)−0.60×exp⁡(−26.34(RT))×t.

Kuşlu et al. investigated ulexite in borax pentahydrate solutions. In order to determine a mathematical model, they used integrated rate equations for the unreacted shrinking core model applying nonlinear regression analysis. The mathematical model was described as follows [10]:

(3) 1−(1−x)1/3=9.725×105×D−0.8×(SL)−0.8×W0.1exp⁡(−42.525(RT))×t.

In (2) and (3) *C*, *W*, *D*, (*S*/*L*), and *t* indicate concentration, stirring speed, mean particle size, solid/liquid ratio, and reaction time, respectively. *x* was designated as dissolution rate for all equations. However, there are some disadvantages of using numerical and statistical methods:

- a large data set is necessary in order to obtain reliable results,
- prediction data cannot accord with experimental data,
- complex calculating is needed,
- being time consuming.

Neural networks are one of the artificial intelligence techniques. It is based on present understanding of the biological nervous system and its ability to learn through example [15, 16]. Neural networks are used to solve the problems which cannot be modeled, in particular. A neural network can learn, adapt, predict, and classify. Prediction of parameters capacity of neural networks is very high. It provides more accurate results than the conventional statistical methods for prediction. Therefore, it has been used in different engineering applications [16–19].

There is nearly no article in the scientific literature specifically devoted to a study of an integrated prediction of dissolution kinetics of boron minerals based on ANNs (see Table 1). In general, in order to determine prediction of dissolution kinetics boron mineral, conventional statistical methods were used by authors [14, 20, 21].

In this study, an artificial neural network was developed to predict dissolution kinetics of colemanite which was determined using the feedforward backpropagation neural network algorithm. ANNs performance was determined by using different learning methods, activation function, hidden layer, and neuron numbers to determine prediction of dissolution rate. ANNs were trained using experimental data [14]. Afterwards, prediction data was obtained from ANNs in comparison with previous mathematical model [14] which was formed using conventional prediction technique. ANNs model has given more accurate results than mathematical model.

This paper is organized as follows. In Section 2, the experimental study and used method are presented. Designing of an artificial neural network for prediction of dissolution rate and simulations method are considered in Section 3. Result of the simulations is addressed in Section 4. Finally, conclusions are presented in Section 5.

## 2. Material and Methods

### 2.1. Material

Dissolution processes were carried out by using colemanite mineral which was obtained from real boric acid plant (*ETI Mine, Bandırma, Turkey*). The samples were crushed by a jaw crusher and sieved using ASTM Standard sieves to obtain the following size fractions; 137.5, 213.5, 446, and 563.5 *μ*m. The chemical characteristics of colemanite minerals, chosen parameters, and their ranges used in experiments are shown in Table 2.

### 2.2. Methods

An artificial neural network was developed to predict dissolution rate of colemanite in this study. Developed neural network results were compared with a regression based mathematical model which was obtained in a previous study. This mathematical model was formed using a series of experiments. Neural network was trained with data obtained from these experiments.

**Figure 1:**
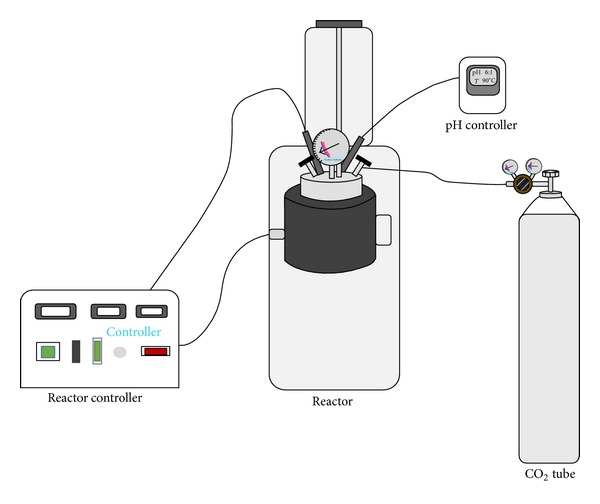
Schematic diagram of the experimental setup.

Experiments were carried out in a reactor (Parr 4848 reactor controller—Parr pressure reactor—Figure 1) which provides the temperature, pressure, stirring speed, and pH control. The schematic diagrams of experimental setup are shown in Figure 1. The solid prepared in accordance with the certain solid/liquid ratio was put into the reactor and 200 mL distilled water was added onto it. The system was set into the desired conditions and after the temperature of the reactor content reached to the determined value, CO_2_ gas was passed through the reactor until the air inside went to the outside. Later the gas outlet valve was closed and after the pressure value was adjusted to the desired level, the experiments were started.

At the end of the reaction, solutions were filtered by using a blue band filter and B_2_O_3_, Na^+^, and Ca^2+^ analyses were measured in permeate by using volumetric method and flame photometry, respectively. The mole fractions of B_2_O_3_, Na_2_O, and CaO passing into the solution from ulexite mineral were calculated by formulas given in (4), where *x* designates quantity dissolution and *i* shows the soluble compound:

(4) $$\begin{array}{c} x_{i}=\frac{\text{Amount}\;\text{of}\;\left(i\right)\;\text{passing}\;\text{into the solution}}{\text{Amount}\;\text{of}\;\left(i\right)\;\text{in original sample}}. \end{array}$$

## 3. Designing of an Artificial Neural Network for Prediction of Dissolution Rate

Artificial neural networks are computational systems that simulate the microstructure (neurons) of a biological nervous system. The most basic components of ANNs are modeled after the structure of the brain, and therefore even the terminology is borrowed from neuroscience [27]. ANNs consist of a large number of processing elements with their interconnections. They are essentially parallel computing systems similar to biological neural networks [28] called neurons, with each layer being fully connected to the proceeding layer by interconnection fully connected to the proceeding layer by interconnection strengths or weights [16].

**Figure 2:**
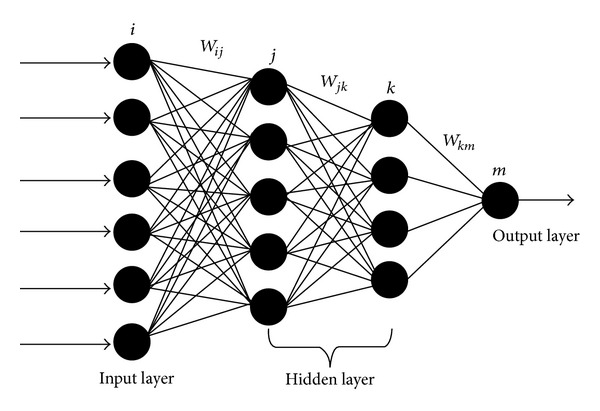
A typical four-layer feedforward ANN.

ANNs are widely used to approximate complex systems that are difficult to model using conventional modeling techniques, such as mathematical modeling. There is no a certain method for selection of proper ANNs structure and training algorithm. The best solution is obtained by trial and error. On the other hand, the neural networks have a high prediction capability. Therefore a neural network, which has multilayer feedforward structure with two hidden layers, was designed (see Figure 2). Pressure, temperature, particle size of colemanite, solid liquid ratio, reaction time, and stirring speed are inputs of ANNs structure. The ANN was trained by using multilayer perceptron (MLP) networks. Backpropagation (BP) algorithm is the typical means of adjusting the weights and biases to obtain minimum the mean square error between the target and the network output by using a gradient descent algorithm [17, 29, 30]. Backpropagation gradient descent to minimize the target error, which is approximated in the vector space created by the weights and biases [31, 32].

In order to implement learning algorithm, each iteration of training includes the following procedures.

(1) Set the initial values of weights *W*_*ij*_ and *W*_*jk*_.

(2) Compute the outputs for all neurons and layer, starting with the input layer as shown below:

(5) $$\begin{array}{c} \text{ne}{\text{t}}_{j}=\overset{I}{\underset{i=1}{\sum }}W_{ij}X_{i},\;j=1,2,\ldots ,J-1,\;\;i=1,2,\ldots ,I,\\ \text{outpu}{\text{t}}_{j}=f\left(\text{ne}{\text{t}}_{j}\right),\\ \text{ne}{\text{t}}_{k}=\overset{J}{\underset{j=1}{\sum }}W_{jk}Y_{j},\;j=1,2,\ldots ,J-1,\;\;k=1,2,\ldots ,K,\\ \text{outpu}{\text{t}}_{k}=f\left(\text{ne}{\text{t}}_{k}\right), \end{array}$$

where *W*_*ij*_ is the weight between the input neurons and the hidden neurons, *W*_*jk*_ is the weight between the hidden and the output nodes, *X*_*i*_ is the value of the input which consists of pressure, temperature, particle size of colemanite, solid liquid ratio, reaction time, and stirring speed, *I* is the number of inputs of neuron *i* in the hidden layer, output_*j*_ is the value of the output for hidden nodes, *j* is the number of neurons of the hidden layer, *J* is the number of inputs of neuron *k* in the output layer, output_*k*_ is the output signals (dissolution of colemanite), and *k* is the number of neurons of the output layer. In order to convert the input signals to the output signals, sigmoid transfer function can be used in ANN. Sigmoid transfer function formula is given below:

(6) $$\begin{array}{c} f\left(x\right)=\frac{1}{1+e^{-\text{net}}}. \end{array}$$

(3) Compute the error. In order to determine ANN performance, root mean square errors (RMSE), mean absolute errors (MAE), and coefficient of correlation (*R* ^2^) parameters were used. These are defined as

(7) RMSE=1N∑i=1N(Yiobserved−Yiestimate)2,MAE=1N∑i=1N|Yiobserved−Yiestimate|,R2=(∑i=1N(Yiobserved−Yi¯observed)2 −∑i=1N(Yiobserved−Yi¯predicted)2)×(∑i=1N(Yiobserved−Yi¯observed)2)−1,

where, ${\overset{-}{Y}}_{\text{measured}}$ is the mean of the measured data [*Y*_measured_, *i*]. RMSE measures residual errors that give a global idea of the difference between the observed and modeled values. And *R* ^2^ provides the variability measure of the data reproduced in the model. ${\bar{Y_{i}}}_{\text{observed}}$ is the average of *Y*_observed_, *i*.

(4) The number of iterations is determined according to minimum value of the error function. When the error function reaches to sufficient value, iteration is stopped.

(5) Learning error computed for every neuron in all layers:

(8) δk=(dk−ak)f′(outputk), k=1,2,…,K,δj=∑k=1KWjkδkf′(outputj),j=1,2,…,J−1, k=1,2,…,K,

where *K* represents the total number of patterns, *δ*_*k*_ the desired outputs (experimental data) and *a*_*k*_ the actual outputs.

(6) Update weights along negative gradient of error:

(9) Wij(n+1)=Wij(n)+lrδk  outputj+α(Wij(n)−Wij(n−1)),Wjk(n+1)=Wjk(n)+lrδj  outputk+α(Wjk(n)−Wjk(n−1)),

where *l*_*r*_ is the learning rate, *α* is the momentum, and *n* is the learning cycle.

(7) These procedures are repeated until the desired value of error.

All calculations were performed with software. The neural network structure is shown in Figure 2. In this figure, *i*, *j*, *k*, and *m* denote nodes input layer, hidden layer, and output layer, respectively. Weight of the nodes is referred to as *w*. Subscripts specify the connections between the nodes. For example, *w*_*ij*_ is the weight between nodes *i* and *j*. The data were randomized and divided into two parts, training and testing. After the randomizing process, 65 data were used for training and 20 data were used for testing. Before applying the ANNs to the data, the training input and output values were normalized using the equation:

(10) $$\begin{array}{c} a\frac{x_{i}-x_{\min}}{x_{\max}-x_{\min}}+b, \end{array}$$

where *x*_min⁡_ and *x*_max⁡_ symbolize the minimum and maximum of the data. Different values can be assigned for the scaling factors *a* and *b*. There are no fixed rules as to which standardization approach should be used in particular circumstances. This range [0.2,0.8] increases the extrapolation ability of the ANN models. Therefore, these factors were assigned as 0.6 and 0.2, respectively [16].

In order to determine the best BP training algorithm, Bayesian regulation, BFGS quasi-Newton methods, and Levenberg-Marquardt (LM) algorithm with one and two hidden layers were trained and validated. Additionally, logsig, tansig, and radbas were used to find the best ANN transfer function. Different combinations of ANN structures with one or two hidden layers are tested in terms of iterations.

The following factors are employed during the training design: constant learning rate, constant moment, and learning cycles. Weights and bias values have been iteratively renewed using the various algorithms to minimize the RMSE between the network output and the target output. These parameters should be preferred to be as small as possible to obtain a best performance from ANN models, since the algorithm goes unstable [31–34].

**Figure 3:**
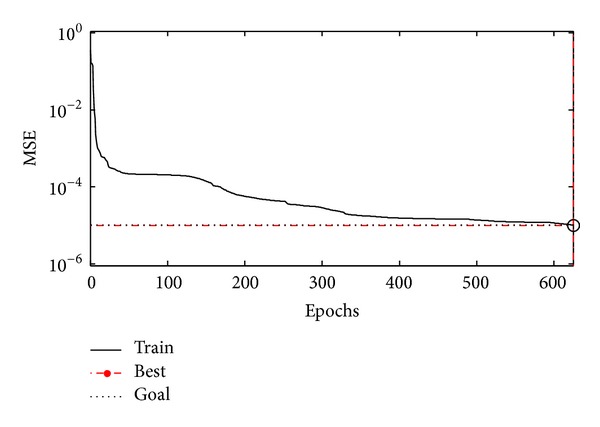
The training error graph for the ANN models.

The ANN networks training was stopped after 625 learning cycles (epochs) since the variation of error was too small after this epoch. MSE is shown in Figure 3 according to epoch. The training was realized using the following parameters:

- constant learning rate = 0.01,
- constant momentum factor = 0.9,
- mean-square error target = 10^−6^.

## 4. Results and Discussion

The performance comparison of BP algorithms in dissolution kinetics of colemanite mineral in water saturated with CO_2_ is given in Table 3. The different structures of ANN models and their outputs are given in Table 3. The performance of ANN outputs for each model was evaluated using three parameters: mean absolute error (MAE), the coefficient of correlation (*R* ^2^), and root mean square errors (RMSE). As can be seen from Table 3,* LM backpropagation* and* logsig* with two hidden layers are more suitable as training and transfer function than others.

**Figure 4:**
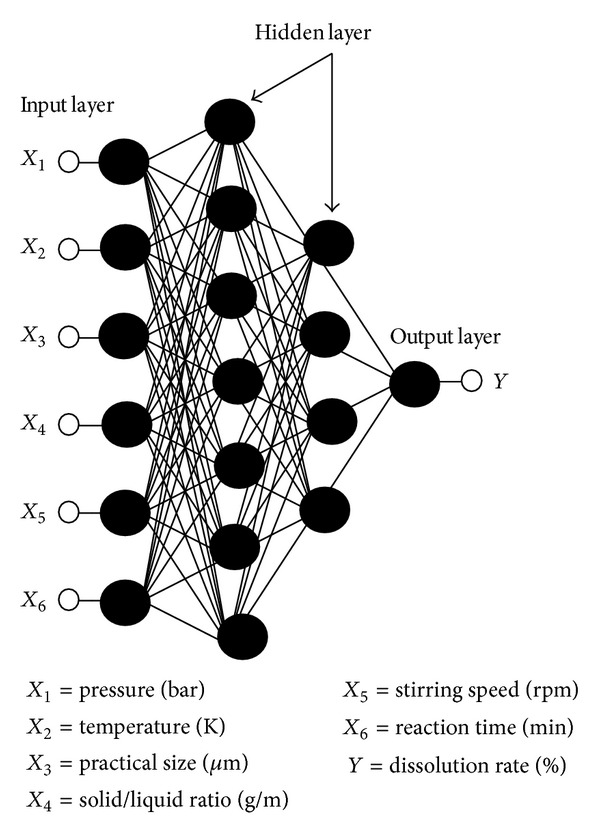
The optimum ANN model structure.

The test RMSE statistics of the ANN models are given in Table 4. The best result for minimum RMSE was selected. In this table, *Y* and *X* represent the number of neurons in the* first* and* second* hidden layers, respectively. As can be seen from this table, the ANN model which has* 7 neurons* in the first hidden layer and* 4 neurons* in the second hidden layer has the lowest RMSE (0.0073), MAE (0.0061), and the highest *R* ^2^ (0.9975) value in test period. According to this result optimum ANN model was determined and structure of it is shown in Figure 4.

**Figure 5:**
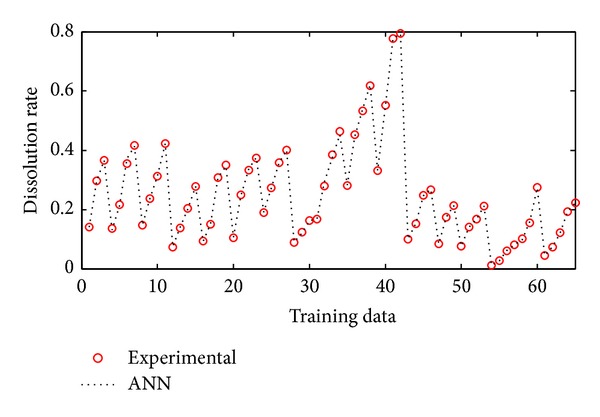
Comparison of ANN and experimental results for the training results.

**Figure 6:**
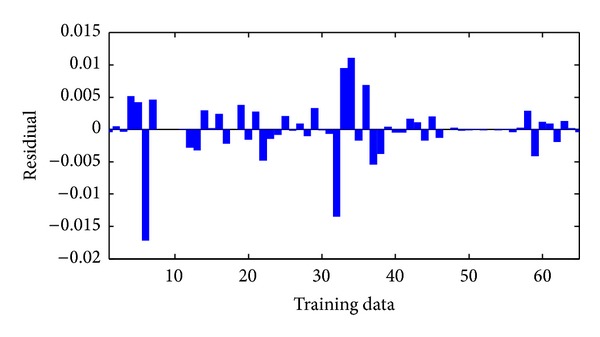
Residuals between ANN and experimental data for training data.

**Figure 7:**
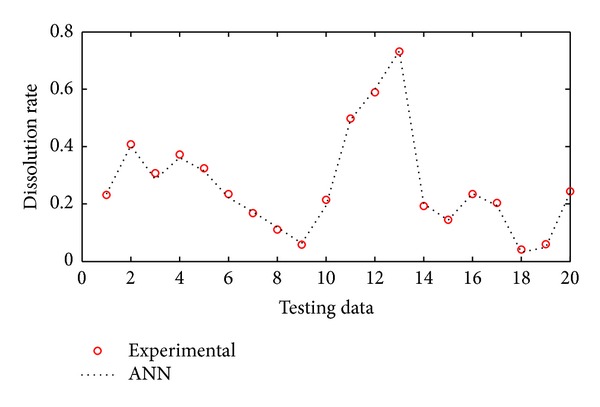
Comparison of ANN and experimental results for the testing results.

**Figure 8:**
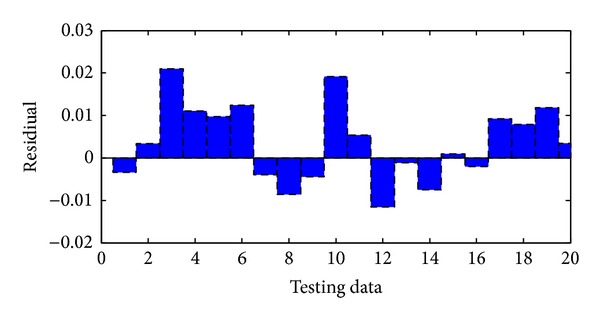
Residual between ANN and experimental for testing data.

The performance of neural networks for training is shown in Figure 5. As can be seen from this figure ANNs results perfectly follow experimental results. The network was evaluated by comparing its predicted output values with experimental data which was shown in Figure 6. As it is seen in the figure the experimental data were found to be compatible with ANNs output. The maximum residual between the experimental data and ann output was determined to be around 0.0017 for training dataset. The residual characteristics have a decreasing trend. After training the neural network, test performance was checked. The performance of test was shown in Figure 7. Figure 7 also shows an analysis between the network outputs (estimations) and the corresponding targets (observed data) for the test data set. It is obvious that the predicted values from the trained neural network outputs catch the targets well. Residuals between testing and experimental data were shown in Figure 8. The maximum residual between estimations and observed data is determined about 0.002 for testing dataset.

**Figure 9:**
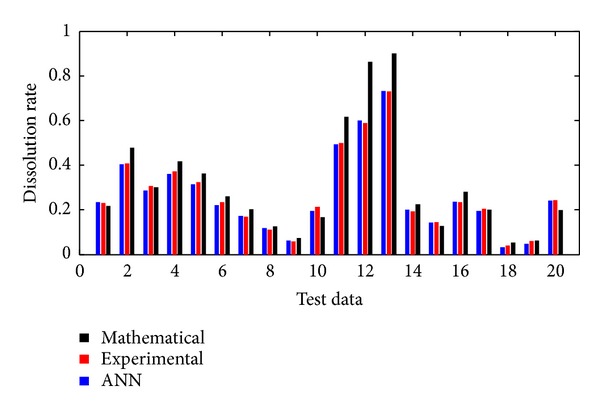
Comparison experimental, mathematical, and ANN for test input.

Çopur et al. used heterogeneous reaction models in order to determine dissolution kinetics. They obtained a mathematical model by using numerical methods based on regression method. This model is given in (1). The dissolution rate was calculated using ANN test inputs with (1). Experimental, mathematical, and ANN results were compared. Results were shown in Figure 9. According to Figure 9 developed ANN results are closer to experimental data than the mathematical model results obtained from numerical methods. As is obvious from Figure 9 ANN gives better results than numerical methods.

## 5. Conclusion

The work presented here has demonstrated that ANN can be successfully employed to predict dissolution kinetics of colemanite mineral. In order to develop ANN models, total pressure, reaction temperature, particle size, solid/liquid ratio, and stirring speed parameters were used as input parameters and dissolution rate as the output. Experimental dataset was used to train multilayer perceptron (MLP) networks to allow for prediction of dissolution kinetics. So as to obtain most suitable prediction data, different learning methods, activation function, hidden layer, and neuron numbers were used. Levenberg-Marquardt backpropagation algorithm and Log-sigmoid (logsig) with two hidden layers were determined as training and transfer function. Also, ANN structure is comprised of 6 input neurons, 7 first hidden, 4 second hidden, and one output layers. This structure has the lowest RMSE (0.0073) and the highest *R* ^2^ (0.9975) values.

Developed ANN has given highly accurate predictions in comparison with an obtained mathematical model used through regression method. We conclude that ANN may be preferred as an alternative approach instead of conventional statistical methods for prediction of boron minerals. The prediction of dissolution kinetics can be obtained using with ANN quickly and accurately.

## Figures and Tables

**Table 1:** Previous studies about dissolution kinetics of boron mineral.

No	Author, year	Mineral	Solution	Method	Reference
1	Guliyev et al. (2012)	Colemanite	Potassium hydrogen sulphate	Analytical numerical	[8]
2	Guliyev et al. (2012)	Colemanite	Ammonium hydrogen sulphate	Analytical numerical	[22]
3	Demirkiran and Künkül (2011)	Ulexite	Ammonium carbonate	Graphical	[23]
4	Kuşlu et al. (2010)	Ulexite	Borax pentahydrate	Nonlinear regression	[10]
5	Çopur et al. (2010)	Colemanite	Water saturated with carbon dioxide	Statistical	[14]
6	Demirkiran (2009)	Ulexite	Ammonium nitrate	Numerical	[24]
7	Ekmekyapar et al. (2008)	Ulexite	Acetic acid	Numerical	[25]
8	Demirkiran (2008)	Ulexite	Ammonium acetate	Statistical	[20]
9	Alkan and Doğan (2004)	Colemanite	Oxalic acid	Statistical	[21]
10	Okur et al. (2002)	Colemanite	Sulfuric acid	Nonlinear regression	[26]
11	**In this study**	Colemanite	Water saturated with carbon dioxide	**ANN**	

**Table 2:** The chemical characteristic of colemanite mineral and ranges of parameters.

Components	Amounts (%)	Parameters	Ranges
B_2_O_3_	42.00	Pressure (bar)	5, 10, 15, 20
CaO	19.75	Temperature (K)	303, 313, 323, 333, 353, 383
H_2_O	18.20	Meanly particle size (µm)	137.5, 213.5, 446, 563.5
SiO_2_	5.54	Solid-liquid ratio (g/mL)	0.10, 0.20, 0.25, 0.30, 0.35
As_2_S_3_	1.22	Stirring speed (rpm)	450, 500, 730
Other	13.22	Reaction time (min.)	2.5, 7.5, 15, 30, 40

**Table 3:** Comparison of various backpropagation algorithms using different transfer function (test).

Training function	Transfer function	One hidden layer			Two hidden layers		
		*R* ^2^	RMSE	MAE	*R* ^2^	RMSE	MAE
LM backpropagation	tansig	0.9933	0.0121	0.0099	0.819	0.0641	0.0374
LM backpropagation	**logsig**	0.9921	0.0135	0.0110	**0.9975**	**0.0073**	**0.0061**
LM backpropagation	radbas	0.4391	0.1320	0.0990	0.5056	0.4184	0.3963
Bayesian regulation backpropagation	tansig	0.9945	0.0113	0.0091	0.9938	0.0120	0.0098
Bayesian regulation backpropagation	logsig	0.9854	0.0167	0.0133	0.9951	0.0102	0.0081
Bayesian regulation backpropagation	radbas	0.9946	0.0117	0.0101	0.9965	0.0090	0.0069
BFGS quasi-Newton backpropagation	tansig	0.9876	0.0167	0.0116	0.9909	0.0136	0.0120
BFGS quasi-Newton backpropagation	logsig	0.9839	0.0189	0.0116	0.9882	0.0150	0.0128
BFGS quasi-Newton backpropagation	radbas	0.0724	0.2041	0.0981	0.5832	0.1397	0.0688

**Table 4:** The RMSE statistics of the ANN models in testing for two hidden layers.

X*	1			2			3			4			5		
Y*	RMSE	MAE	R^2^	RMSE	MAE	R^2^	RMSE	MAE	R^2^	RMSE	MAE	R^2^	RMSE	MAE	R^2^
1	0.0320	0.0245	0.962	0.0319	0.0245	0.9616	0.0629	0.0486	0.7928	0.0469	0.0312	0.8822	0.0477	0.0323	0.8852
2	0.0187	0.0168	0.9814	0.0236	0.0186	0.97	0.4184	0.3963	NaN	0.0171	0.014	0.9868	0.0191	0.0127	0.9801
3	0.0229	0.0151	0.9836	0.0154	0.0121	0.987	0.0129	0.0101	0.9939	0.1442	0.0456	0.195	0.0179	0.0157	0.9828
4	0.0268	0.0215	0.9609	0.0107	0.0089	0.9945	0.0322	0.0222	0.943	0.0902	0.0366	0.592	0.0219	0.0144	0.9749
5	0.0143	0.0114	0.9905	0.0105	0.0082	0.9961	0.0228	0.0174	0.9714	0.0249	0.0175	0.9717	0.0277	0.0183	0.9588
6	0.0147	0.01	0.989	0.0142	0.0106	0.9923	0.0285	0.0145	0.9627	0.0217	0.0158	0.9811	0.022	0.0161	0.98
7	0.0286	0.0147	0.9601	0.017	0.0135	0.9852	0.0156	0.0093	0.9893	**0.0073**	**0.0061**	**0.9975**	0.0159	0.0133	0.9904
8	0.0177	0.0118	0.9836	0.0281	0.0188	0.9565	0.0889	0.048	0.8232	0.0219	0.015	0.9809	0.07	0.037	0.7685
9	0.0282	0.0213	0.9632	0.0313	0.0194	0.9495	0.0263	0.0168	0.9831	0.0575	0.0464	0.8877	0.0275	0.0192	0.9686
10	0.0226	0.0171	0.9729	0.0533	0.0352	0.8669	0.0418	0.0192	0.9618	0.0193	0.0132	0.9874	0.0179	0.0134	0.9826

X*	6			7			8			9			10		
Y*	RMSE	MAE	R^2^	RMSE	MAE	R^2^	RMSE	MAE	R^2^	RMSE	MAE	R^2^	RMSE	MAE	R^2^

1	0.0716	0.0436	0.8371	0.0712	0.0447	0.8416	0.0544	0.0452	0.8401	0.0449	0.0325	0.8908	0.044	0.0355	0.8941
2	0.0387	0.0258	0.9375	0.1006	0.0349	0.5589	0.0757	0.0508	0.861	0.0904	0.0536	0.6878	0.1476	0.0808	0.3663
3	0.1158	0.0432	0.6194	0.0459	0.0268	0.9042	0.0193	0.0151	0.9813	0.0301	0.0222	0.9503	0.1174	0.0814	0.4844
4	0.0385	0.0244	0.9406	0.1053	0.0548	0.8877	0.0779	0.035	0.7952	0.1249	0.0671	0.5727	0.0387	0.0254	0.9448
5	0.0684	0.0385	0.7699	0.0157	0.0129	0.9885	0.0145	0.0116	0.9905	0.4184	0.3963	0.0723	0.0298	0.0166	0.9697
6	0.0352	0.0235	0.939	0.0908	0.0489	0.8513	0.1306	0.0529	0.5611	0.0435	0.0187	0.9046	0.0291	0.0225	0.9597
7	0.0307	0.0188	0.9514	0.0136	0.0118	0.9925	0.0497	0.0223	0.8686	0.0365	0.0236	0.9419	0.0129	0.0102	0.9926
8	0.0162	0.012	0.9874	0.0368	0.0273	0.9329	0.0349	0.0257	0.9443	0.0279	0.0185	0.9744	0.0154	0.0131	0.9874
9	0.0436	0.029	0.8985	0.0462	0.0304	0.9288	0.0458	0.024	0.892	0.0225	0.0184	0.9732	0.039	0.0308	0.9325
10	0.0318	0.0195	0.9712	0.0424	0.02	0.9675	0.0275	0.0185	0.9594	0.0243	0.0186	0.9696	0.051	0.0225	0.8714

*X: number of nodes in the second hidden layer. *Y*: number of nodes in the first hidden layer.
